# Supplementary material for: An integrated chromatin accessibility and transcriptome landscape of human pre-implantation embryos
Source: Nat Commun. 2019 Jan 21;10:364. doi: 10.1038/s41467-018-08244-0 (PMC6341076; doi:10.1038/s41467-018-08244-0)
Supplement: Supplementary file 1 — Supplementary Information [file 41467_2018_8244_MOESM1_ESM.pdf]

An integrated chromatin accessibility and transcriptome landscape of human pre-implantation embryos

Liu *et al.*

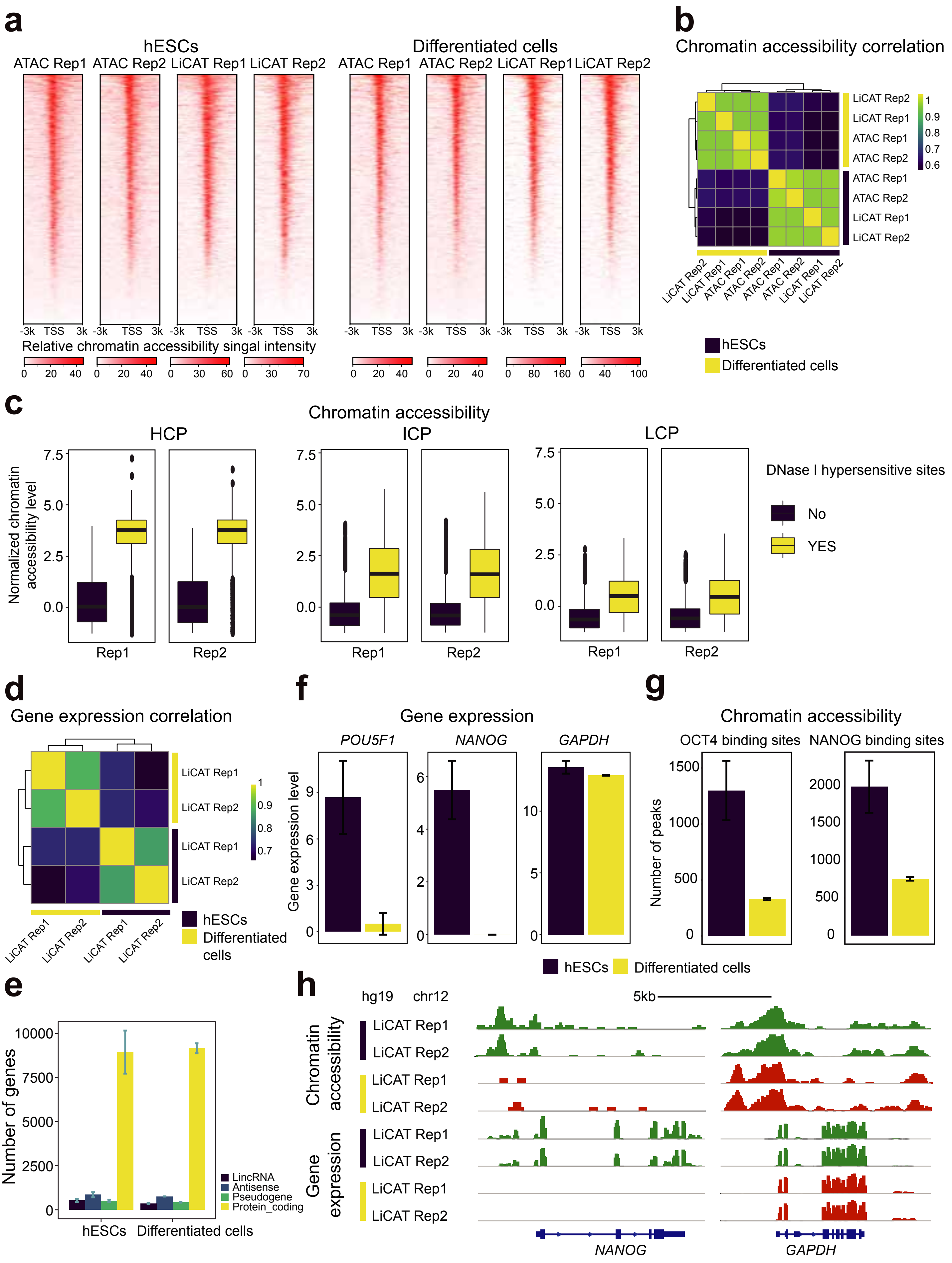

a
hESCs
Differentiated cells
ATAC Rep1
ATAC Rep2
LiCAT Rep1
LiCAT Rep2
-3k TSS 3k
Relative chromatin accessibility singal intensity
b
Chromatin accessibility correlation
hESCs
Differentiated cells
c
Chromatin accessibility
HCP
ICP
LCP
Normalized chromatin accessibility level
DNase I hypersensitive sites
No
YES
Rep1
Rep2
d
Gene expression correlation
hESCs
Differentiated cells
f
Gene expression
POU5F1
NANOG
GAPDH
Gene expression level
g
Chromatin accessibility
OCT4 binding sites
NANOG binding sites
Number of peaks
hESCs
Differentiated cells
e
Number of genes
LincRNA
Antisense
Pseudogene
Protein_coding
hESCs
Differentiated cells
h
hg19
chr12
5kb
Chromatin accessibility
Gene expression
LiCAT Rep1
LiCAT Rep2
NANOG
GAPDH

Supplementary Figure 1: Quality evaluation of LiCAT-seq generated chromatin accessibility and gene expression datasets from hESCs and differentiated cells. (a) Heat maps showing the enrichment of chromatin accessibility reads around TSSs in LiCAT-seq profiles generated from 10 cells and ATAC-seq profiles generated from 50,000 cells. **(b)** Pearson correlations between the indicated chromatin accessibility profiles. **(c)** The chromatin accessibility signal density on the HCPs, ICPs and LCPs at the indicated stages. The HCPs, ICPs and LCPs are divided into two groups based on their DNase I hypersensitivity. The DNase I hypersensitive site dataset was downloaded from AnnotationHub (ID: AH22507). **(d)** Pearson correlations between the indicated gene expression profiles. **(e)** Number of genes detected in LiCAT-seq profiles of 4-cell and morula stages of mouse embryo development. **(f)** Number of chromatin accessibility peaks on NANOG and OCT4 binding sites in hESCs and differentiated cells. The NANOG and OCT4 binding sites in hESCs were downloaded from AnnotationHub (NANOG ID: AH22662; OCT4 ID: AH22667). **(g)** The expression level of *NANOG*, *OCT4* and *GAPDH* in hESCs and differentiated cells. **(h)** Genome browser views showing the chromatin accessibility and gene expression signal around *NANOG* and *GAPDH* genes. The error bars shown in this figure represent the mean ± standard deviation (SD) of two replicates.

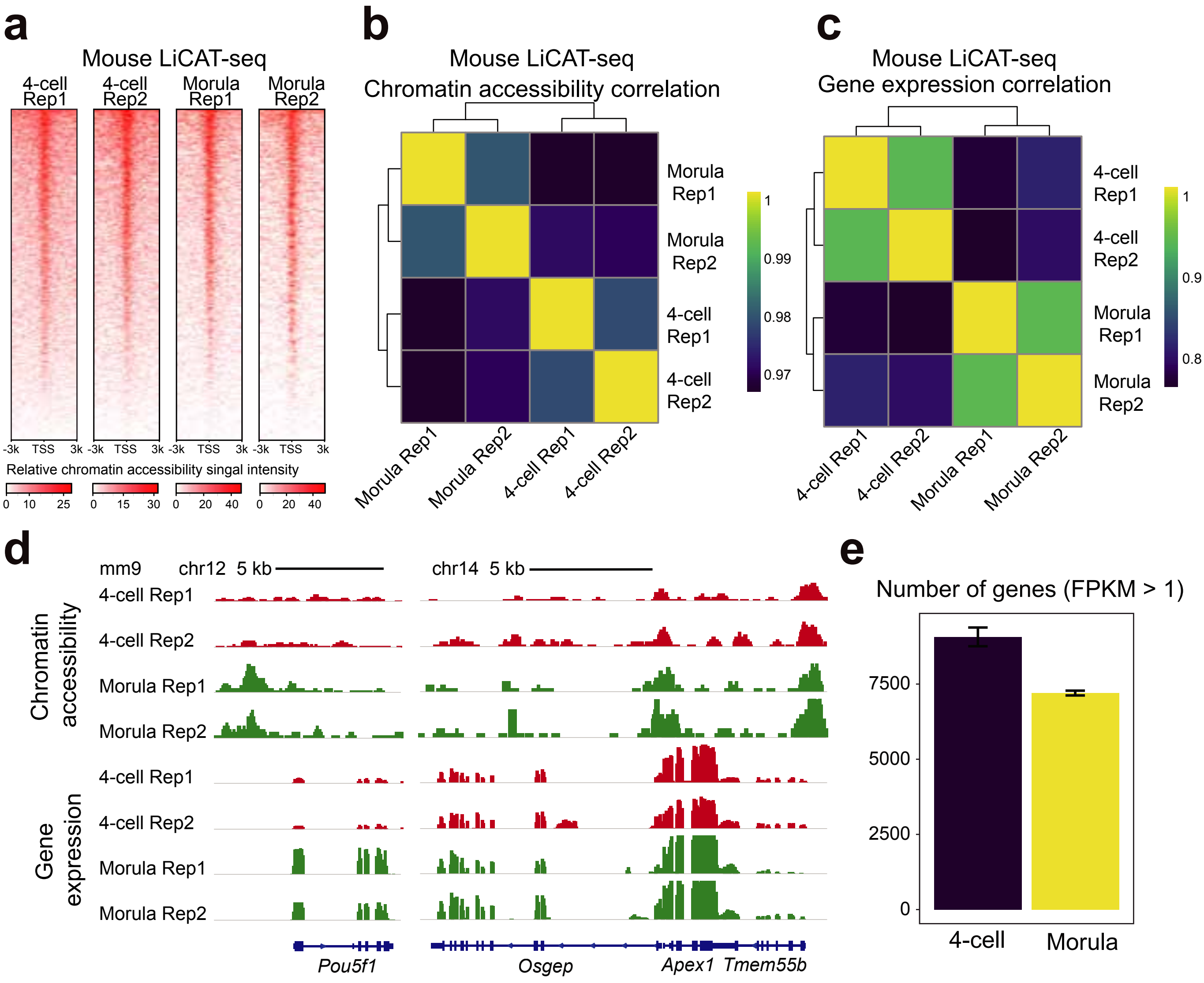

Supplementary Figure 2: Quality evaluation of LiCAT-seq generated chromatin accessibility and gene expression datasets from mouse embryos. (a) Heat maps showing the enrichment of chromatin accessibility reads around TSSs in LiCAT-seq profiles generated from 4-cell and morula stages of mouse embryo development. **(b)** Pearson correlations between the indicated chromatin accessibility profiles. **(c)** Pearson correlations between the indicated gene expression profiles. **(d)** Genome browser views showing the chromatin accessibility and gene expression signal around Oct4 and Osgep genes. **(e)** Number of genes detected in LiCAT-seq profiles of 4-cell and morula stages of mouse embryo development. The error bars shown in this figure represent the mean ± standard deviation (SD) of two replicates.

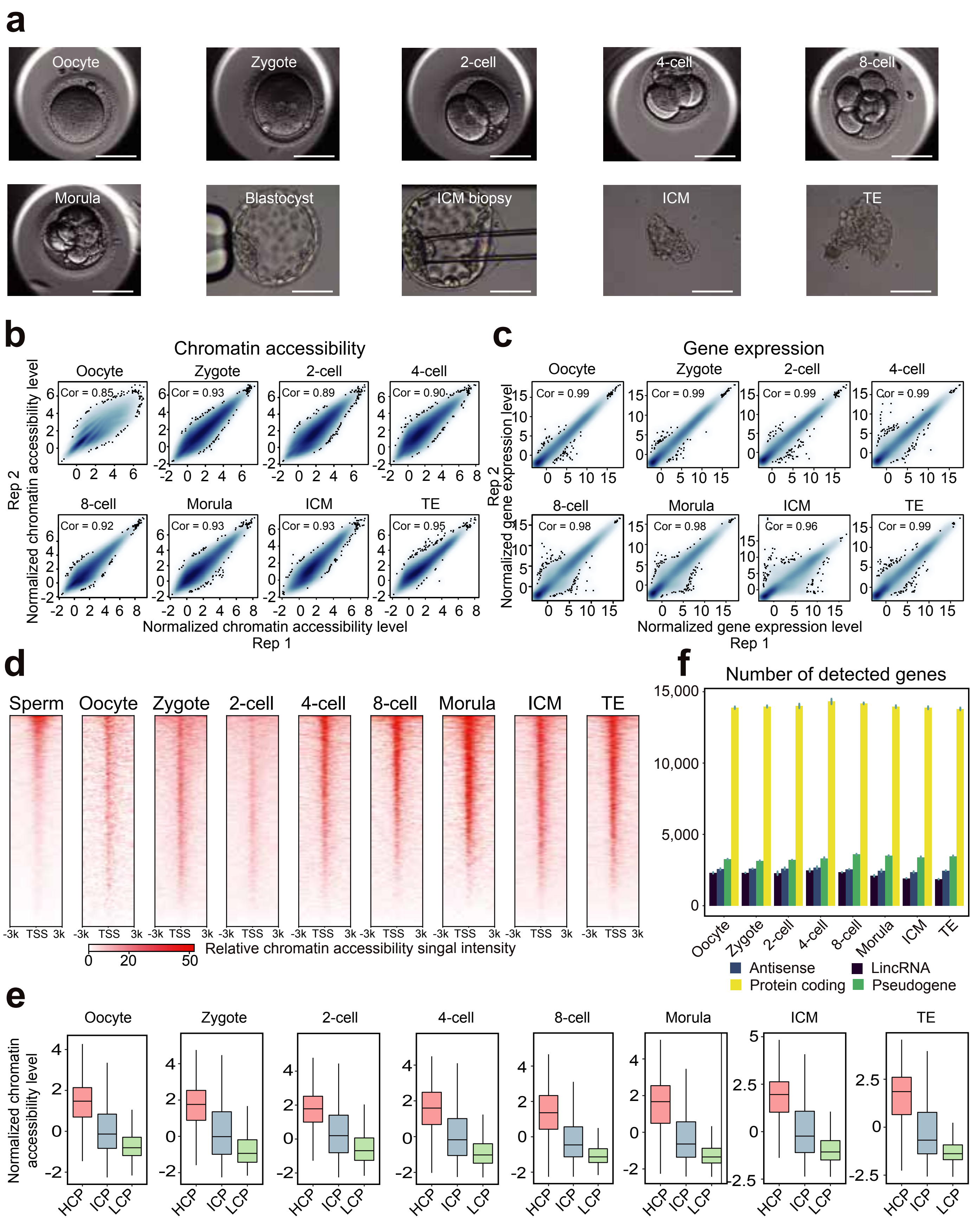
a
Oocyte
Zygote
2-cell
4-cell
8-cell
Morula
Blastocyst
ICM biopsy
ICM
TE
b
Chromatin accessibility
Oocyte
Zygote
2-cell
4-cell
8-cell
Morula
ICM
TE
Cor = 0.85
Cor = 0.93
Cor = 0.89
Cor = 0.90
Cor = 0.92
Cor = 0.93
Cor = 0.93
Cor = 0.95
Rep 2
Normalized chromatin accessibility level
Normalized chromatin accessibility level
Rep 1
c
Gene expression
Cor = 0.99
Cor = 0.99
Cor = 0.99
Cor = 0.99
Cor = 0.98
Cor = 0.98
Cor = 0.96
Cor = 0.99
Rep 2
Normalized gene expression level
Normalized gene expression level
Rep 1
d
Sperm
Oocyte
Zygote
2-cell
4-cell
8-cell
Morula
ICM
TE
-3k TSS 3k
Relative chromatin accessibility singal intensity
0
20
50
f
Number of detected genes
15,000
10,000
5,000
0
Antisense
LincRNA
Protein coding
Pseudogene
e
Normalized chromatin accessibility level
HCP
ICP
LCP

Supplementary Figure 3: Quality assessment of chromatin accessibility and gene expression profiles of human preimplantation embryos. (a) Microscopy imaging of mature human oocytes and preimplantation embryos at the zygote, 2-cell, 4-cell, 8-cell, morula and blastocyst stages (isolated inner cell mass (ICM) and trophectoderm (TE) cells). The scale bars on images of Oocyte, Zygote, 2-cell, 4-cell, 8-cell, Morula, Blastocyst and ICM biopsy represent 50 μm; The scale bars on images of ICM and TE represent 100 μm. **(b)** and **(c)** Scatterplots showing high correlation of chromatin accessibility **(b)** and gene expression **(c)** levels between the two biological replicates of LiCAT-seq profiles at the indicated stages. **(d)** Heat maps showing the enrichment of chromatin accessibility reads around TSSs in each developmental stage. **(e)** The chromatin accessibility signal density on the HCPs, ICPs and LCPs at the indicated stages. **(f)** Number of genes detected at the indicated stages. The error bars shown in this figure represent the mean ± standard deviation (SD) of two replicates.

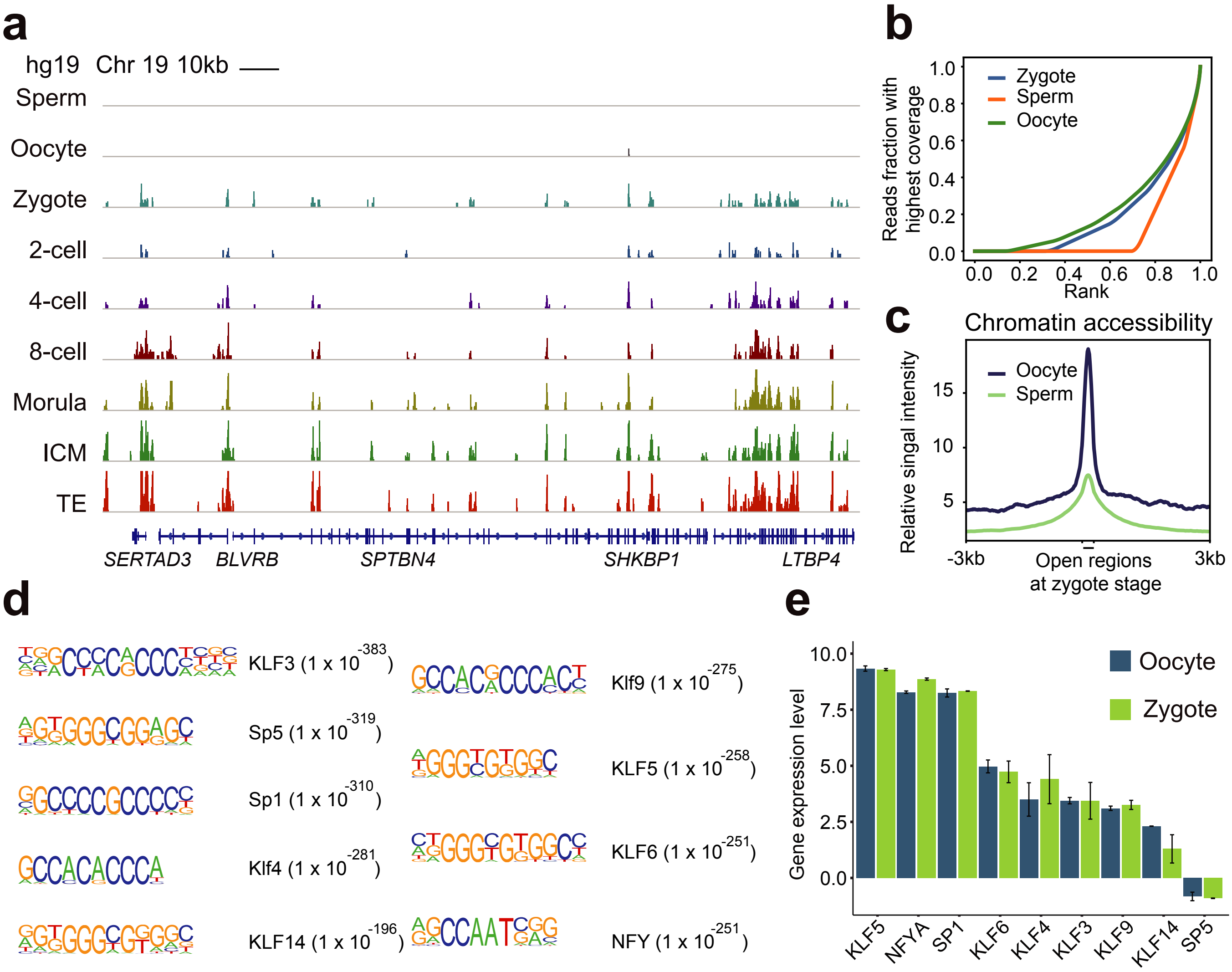

Supplementary Figure 4: Unique chromatin accessibility features at zygote stage. (a) Genome browser view showing the chromatin accessibility peaks within a constitutive accessible region. **(b)** Cumulative sum of regions with different ranks of sequencing coverage showing the enrichment of chromatin accessibility read in specific regions. **(c)** Relative read density around open regions of the zygote stage. **(d)** Enrichment of the indicated transcription factor motifs found within open regions of the zygote stage. **(e)** Expression level of the indicated TFs (shown in **d**) in the oocyte. The error bars shown in this figure represent the mean ± standard deviation (SD) of two replicates.

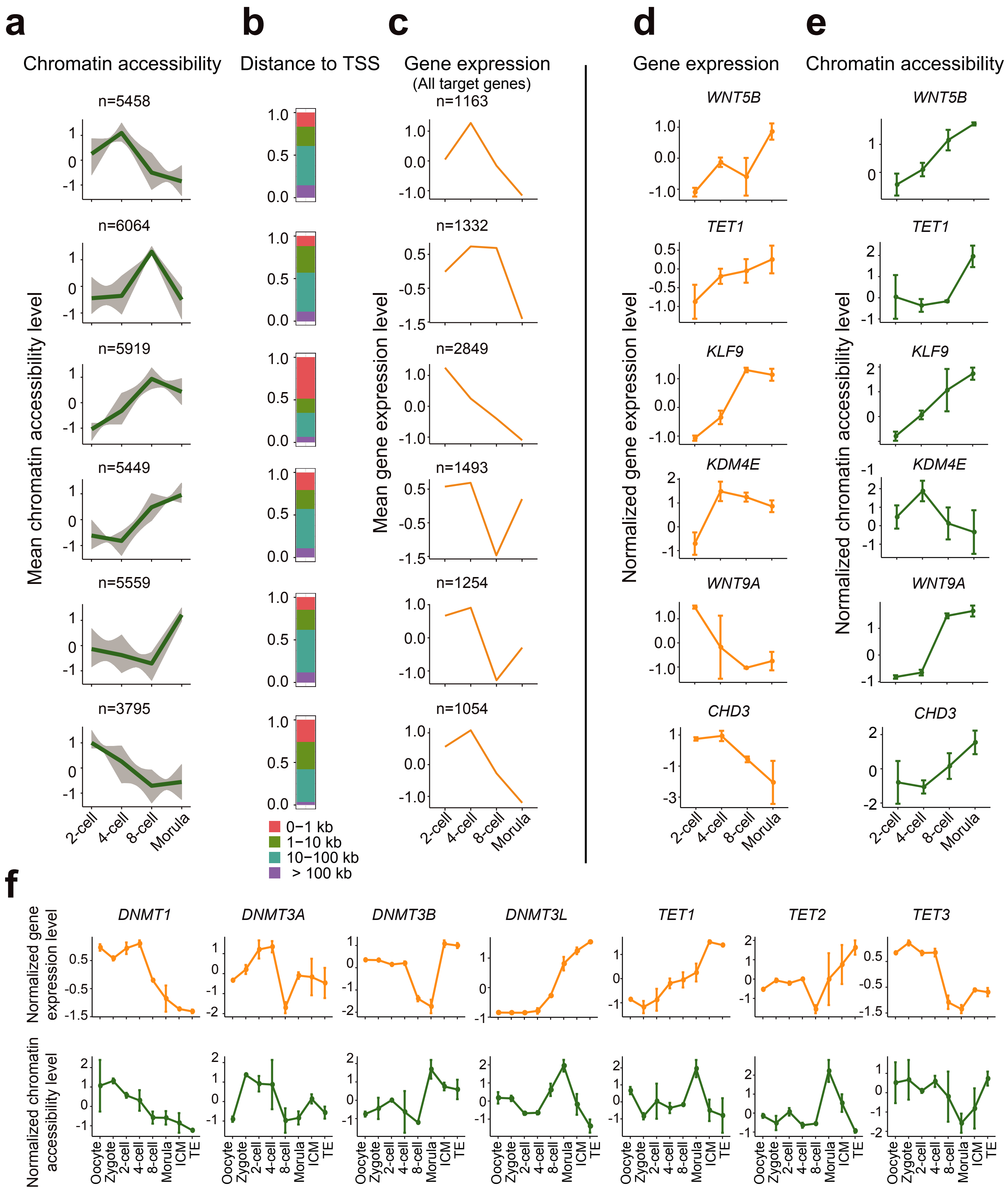

a
b
c
d
e
Chromatin accessibility
Distance to TSS
Gene expression
(All target genes)
Gene expression
Chromatin accessibility
Mean chromatin accessibility level
Mean gene expression level
Normalized gene expression level
Normalized chromatin accessibility level
n=5458
n=6064
n=5919
n=5449
n=5559
n=3795
n=1163
n=1332
n=2849
n=1493
n=1254
n=1054
WNT5B
TET1
KLF9
KDM4E
WNT9A
CHD3
2-cell
4-cell
8-cell
Morula
0−1 kb
1−10 kb
10−100 kb
> 100 kb
f
DNMT1
DNMT3A
DNMT3B
DNMT3L
TET1
TET2
TET3
Normalized gene expression level
Normalized chromatin accessibility level
Oocyte
Zygote
2-cell
4-cell
8-cell
Morula
ICM
TE

Supplementary Figure 5: Dynamics of chromatin accessibility and gene expression during embryonic genome activation. (a) Fuzzy clustering analysis of chromatin accessibility signals for the indicated stages. The individual grey lines represent the accessibility level of individual regions, and the green line represents the value for the cluster center. **(b)** The distribution of distances to the nearest TSS for regions in each cluster in **(a)**. **(c)** Mean standardized gene expression for all putative target genes for regions in each cluster in **(a)**. **(d)** and **(e)** The dynamics of gene expression **(d)** and chromatin accessibility **(e)** for representative genes in each cluster in Fig. 2a. **(f)** The dynamics of gene expression (top) and chromatin accessibility (bottom) for the indicated genes. The error bars shown in this figure represent the mean ± standard deviation (SD) of two replicates.

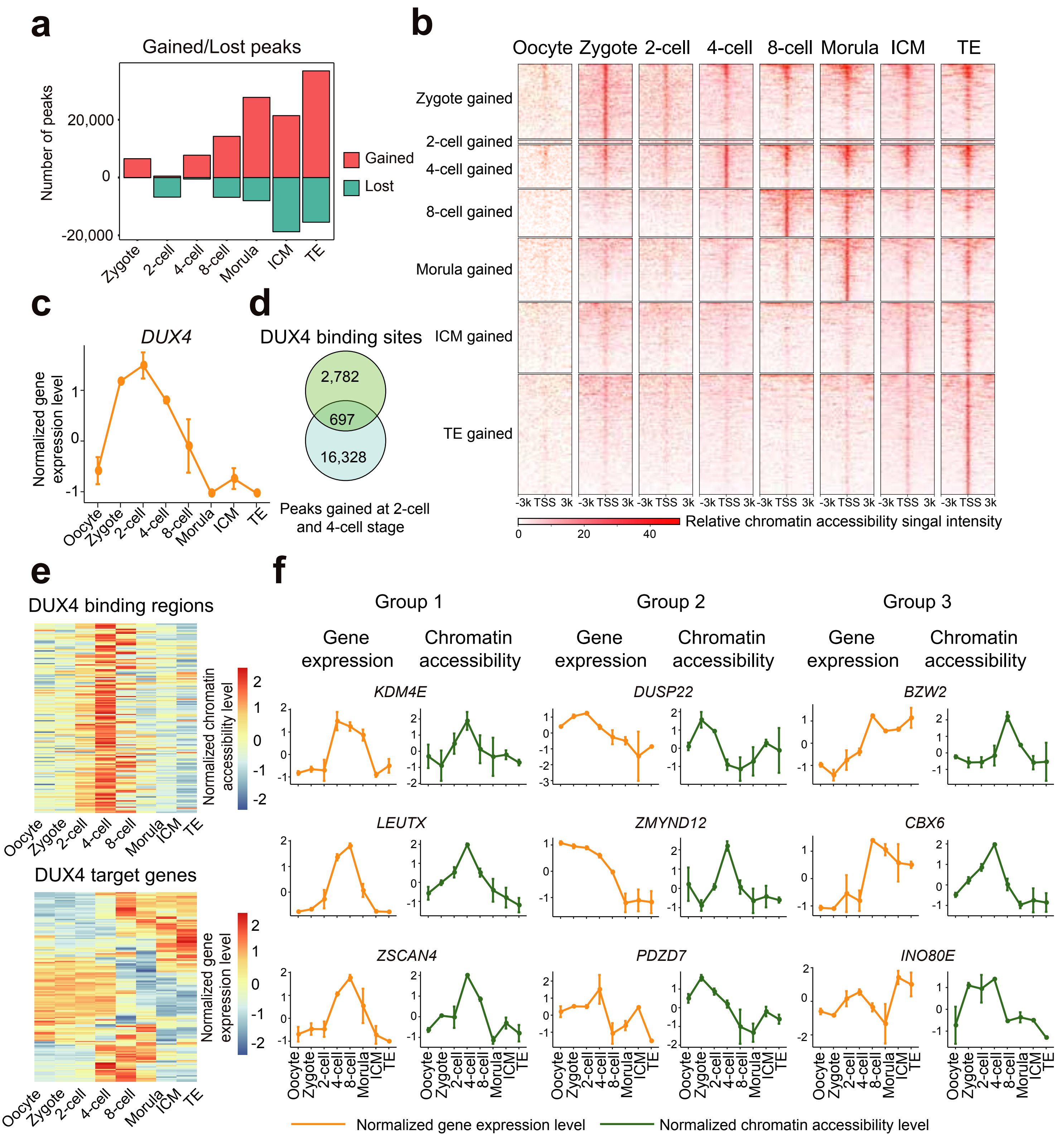
a
Gained/Lost peaks
Number of peaks
20,000
0
-20,000
Gained
Lost
Zygote
2-cell
4-cell
8-cell
Morula
ICM
TE
b
Oocyte Zygote 2-cell 4-cell 8-cell Morula ICM TE
Zygote gained
2-cell gained
4-cell gained
8-cell gained
Morula gained
ICM gained
TE gained
-3k TSS 3k
Relative chromatin accessibility singal intensity
0 20 40
c
DUX4
Normalized gene expression level
Oocyte Zygote 2-cell 4-cell 8-cell Morula ICM TE
d
DUX4 binding sites
2,782
697
16,328
Peaks gained at 2-cell and 4-cell stage
e
DUX4 binding regions
Normalized chromatin accessibility level
DUX4 target genes
Normalized gene expression level
f
Group 1
Group 2
Group 3
Gene expression
Chromatin accessibility
KDM4E
DUSP22
BZW2
LEUTX
ZMYND12
CBX6
ZSCAN4
PDZD7
INO80E
Normalized gene expression level
Normalized chromatin accessibility level

Supplementary Figure 6: DUX4 is an early regulator during embryonic genome activation. **(a)** Number of gained and lost chromatin accessibility peaks at the indicated stages. **(b)** Heat maps showing the chromatin accessibility read enrichment on the gained accessible regions at the indicated stages. **(c)** *DUX4* gene expression level at the indicated stages. **(d)** Venn plot showing the overlap between DUX4 binding sites and accessible regions that are gained at the 2-cell and 4-cell stages. **(e)** Upper panel: heat maps showing the chromatin accessibility level at the indicated stages on DUX4 binding regions that are accessible at 2-cell and 4-cell stages. Bottom panel: heat maps showing the gene expression level of the putative target genes of DUX4 at the indicated stages. **(f)** The dynamics of chromatin accessibility and gene expression for the representative genes in each category. The error bars shown in this figure represent the mean ± standard deviation (SD) of two replicates.

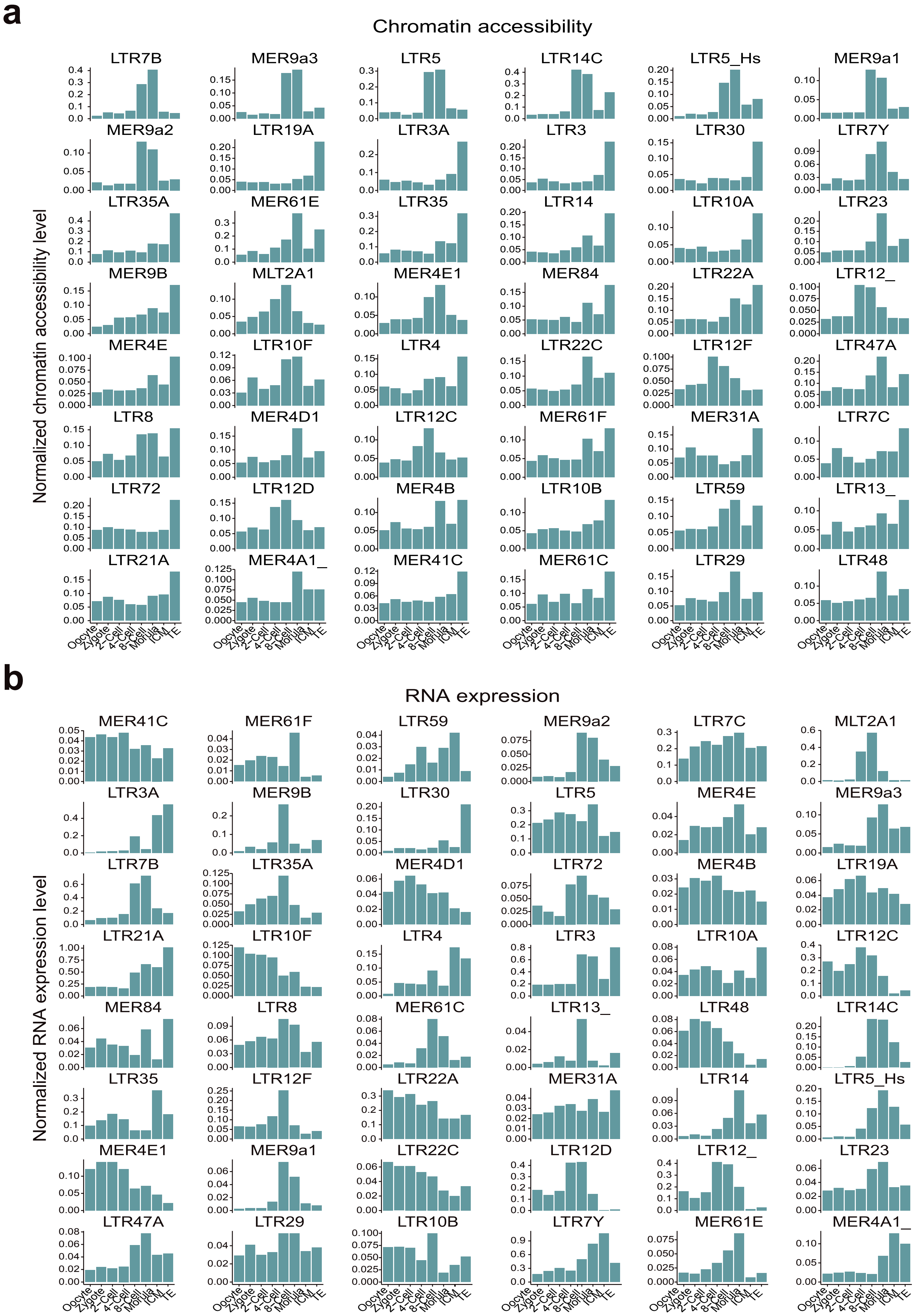
a
Chromatin accessibility
Normalized chromatin accessibility level
LTR7B MER9a3 LTR5 LTR14C LTR5_Hs MER9a1
MER9a2 LTR19A LTR3A LTR3 LTR30 LTR7Y
LTR35A MER61E LTR35 LTR14 LTR10A LTR23
MER9B MLT2A1 MER4E1 MER84 LTR22A LTR12_
MER4E LTR10F LTR4 LTR22C LTR12F LTR47A
LTR8 MER4D1 LTR12C MER61F MER31A LTR7C
LTR72 LTR12D MER4B LTR10B LTR59 LTR13_
LTR21A MER4A1_ MER41C MER61C LTR29 LTR48
Oocyte Zygote 2-Cell 4-Cell 8-Cell Morula ICM TE
b
RNA expression
Normalized RNA expression level
MER41C MER61F LTR59 MER9a2 LTR7C MLT2A1
LTR3A MER9B LTR30 LTR5 MER4E MER9a3
LTR7B LTR35A MER4D1 LTR72 MER4B LTR19A
LTR21A LTR10F LTR4 LTR3 LTR10A LTR12C
MER84 LTR8 MER61C LTR13_ LTR48 LTR14C
LTR35 LTR12F LTR22A MER31A LTR14 LTR5_Hs
MER4E1 MER9a1 LTR22C LTR12D LTR12_ LTR23
LTR47A LTR29 LTR10B LTR7Y MER61E MER4A1_
Oocyte Zygote 2-Cell 4-Cell 8-Cell Morula ICM TE

Supplementary Figure 7: Chromatin accessibility and expression dynamics of endogenous retroviral elements during human preimplantation development. **(a)** Chromatin accessibility level of the indicated ERV elements at the indicated stages. **(b)** RNA expression level of the indicated ERV elements at the indicated stages.

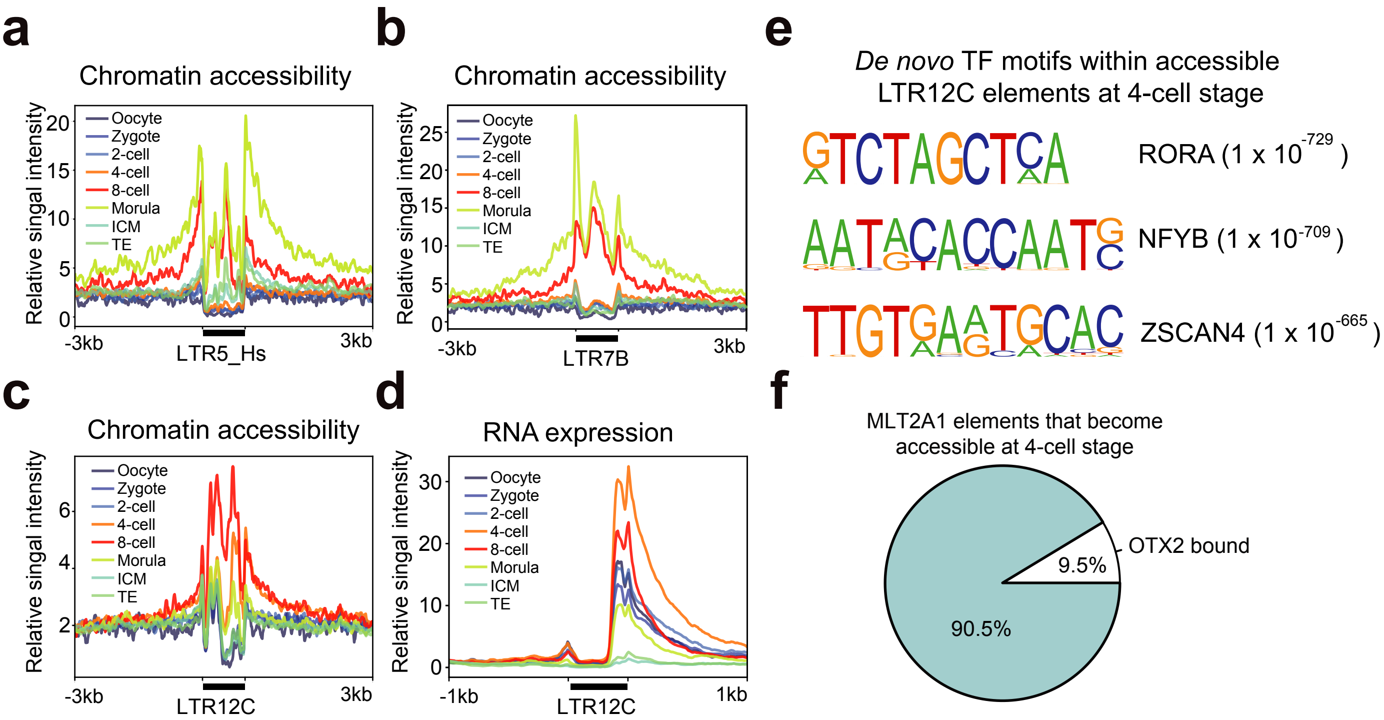

Supplementary Figure 8: Chromatin accessibility, RNA expression, and TF enrichment analysis of the key ERV elements. (a) and **(b)** Chromatin accessibility read enrichment around LTR5_Hs **(a)** and LTR7B **(b)** elements at the indicated stages. **(c)** and **(d)** Chromatin accessibility **(c)** and RNA **(d)** read enrichment around LTR12C elements at the indicated stages. **(e)** The enrichment of the indicated transcription factor binding motifs found within LTR12C elements that become accessible at the 4-cell stage. **(f)** Proportion of MLT2A1 elements that are bound by OTX2 within all MLT2A1 elements that become accessible at the 4-cell stage.

Supplementary Table 1 Summary of the embryo and cell samples used in this study.

	Stages/Samples	Biological replicate	No. of input embryos of each replicate	No. of input cells of each replicate	Donor ID
Human	MII_Oocyte	Rep.1	10 oocytes	10 cells	patient #1, #2
		Rep.2	10 oocytes	10 cells	patient #3, #4
	Sperm	Rep.1	15 sperms	15 cells	patient #5
		Rep.2	15 sperms	15 cells	patient #6
	Zygote	Rep.1	10 embryos	10 cells	patient #7, #8, #9
		Rep.2	9 embryos	9 cells	patient #10, #11
	2-cell	Rep.1	5 embryos	10 cells	patient #12, #13
		Rep.2	5 embryos	10 cells	patient #15, #16
	4-cell	Rep.1	3 embryos	12 cells	patient #17, #18
		Rep.2	3 embryos	12 cells	patient #19
	8-cell	Rep.1	2 embryos	15 cells	patient # 20
		Rep.2	2 embryos	16 cells	patient #21, #22
	Morula	Rep.1	1 embryos	17 cells	patient # 23
		Rep.2	1 embryos	15 cells	patient # 24
	Inner cell mass(ICM)	Rep.1	1 embryos	10 cells	patient # 25
		Rep.2	1 embryos	9 cells	patient # 26
	Trophectoderm cells (TE)	Rep.1	1 embryos	15 cells	patient # 25
		Rep.2	1 embryos	14 cells	patient # 26
Mouse	4-cell	Rep.1	3 embryos	12 cells	-
		Rep.2	3 embryos	10 cells	-
	Morula	Rep.1	1 embryos	13 cells	-
		Rep.2	1 embryos	14 cells	-
hESCs	hESCs-LiCAT	Rep.1	-	15 cells	-
		Rep.2	-	15 cells	-
	hESCs-ATAC	Rep.1	-	~ 5×10^4 cells	-
		Rep.2	-	~ 5×10^4 cells	-
Differentiated cells	Differentiated cells-LiCAT	Rep.1	-	15 cells	-
		Rep.2	-	15 cells	-
	Differentiated cells-ATAC	Rep.1	-	~ 5×10^4 cells	-
		Rep.2	-	~ 5×10^4 cells	-

Supplementary Table 2 Primers used in this study.

Oligo-dT primer	5'-AAGCAGTGGTATCAACGCAGAGTACT$_{30}$VN-3', where V is either A, C or G, and N is any base
Template-Switching Oligo	5'-AAGCAGTGGTATCAACGCAGAGTACATrGrG+G-3', where "r" indicates a ribonucleic acid base and "+" indicates a locked nucleic acid base
PCR primer	5'-AAGCAGTGGTATCAACGCAGAGT-3'
Transposase adapter 1	5'-TCGTCGGCAGCGTCAGATGTGTATAAGAGACAG-3'
Transposase adapter 2	5'-GTCTCGTGGGCTCGGAGATGTGTATAAGAGACAG-3'
