# Supplementary material for: An integrated chromatin accessibility and transcriptome landscape of human pre-implantation embryos
Source: Nat Commun. 2019 Jan 21;10:364. doi: 10.1038/s41467-018-08244-0 (PMC6341076; doi:10.1038/s41467-018-08244-0)
Supplement: Supplementary file 2 — Description of Additional Supplementary Files [file 41467_2018_8244_MOESM2_ESM.pdf]

Description of Additional Supplementary Files

File Name: Supplementary Data 1
Description: Summary statistics of all sequencing datasets used in this study.

File Name: Supplementary Data 2
Description: Normalized chromatin accessibility and gene expression level of EGA genes at each stage.

File Name: Supplementary Data 3
Description: Enrichment of TF motifs at each stage.

File Name: Supplementary Data 4
Description: Normalized chromatin accessibility and RNA expression level of retrotransposons at each stage.
